# An endoplasmic reticulum stress- and Golgi apparatus-related signature reveals immune microenvironment remodeling and *MUC16*-driven PI3K/AKT activation in lung adenocarcinoma

**DOI:** 10.3389/fimmu.2026.1887786

**Published:** 2026-09-08

**Authors:** Weihao Zhang, Lei Liu, Jianwei Liu

**Affiliations:** 1 Graduate School, Chengde Medical University, Chengde, Hebei, China; 2 Department of Cardiothoracic Surgery, Chengde Central Hospital, Chengde, Hebei, China

**Keywords:** endoplasmic reticulum stress, Golgi apparatus, lung adenocarcinoma, *MUC16*, prognostic signature, tumor immune microenvironment

## Abstract

**Background:**

Lung adenocarcinoma (LUAD), the most prevalent histological subtype of non-small cell lung cancer (NSCLC), is characterized by substantial clinical heterogeneity and a frequent propensity to acquire resistance to targeted therapies. Accumulating evidence indicates that both endoplasmic reticulum stress and Golgi apparatus dysfunction play critical roles in reshaping the tumor microenvironment (TME). However, their coordinated contribution to LUAD progression remains poorly understood. Against this background, we developed a machine learning-based prognostic framework focused on endoplasmic reticulum stress- and Golgi apparatus-related genes (EGRGs) to improve prognostic stratification and explore their potential therapeutic implications in LUAD.

**Methods:**

Publicly available transcriptomic profiles and corresponding clinical data of LUAD patients were collected from TCGA and GEO. DEGs identified in the TCGA-LUAD cohort were intersected with endoplasmic reticulum stress- and Golgi apparatus-related genes (EGRGs) to obtain differentially expressed EGRGs in LUAD. A machine learning-based prognostic signature was constructed in the TCGA-LUAD cohort and validated in external GEO datasets. Patients were stratified according to the calculated risk score, after which tumor mutational burden, immune microenvironment characteristics, and drug sensitivity were compared between risk groups. In addition, the key gene *MUC16* was further evaluated through *in vitro* experiments.

**Results:**

A 17-gene prognostic signature was established based on 133 differentially expressed endoplasmic reticulum stress- and Golgi apparatus-related genes. The prognostic performance of this signature was further supported in additional independent LUAD cohorts, and the defined risk groups differed significantly in tumor microenvironment features, immune infiltration patterns, mutational landscapes, and predicted therapeutic responses. Subsequent bioinformatic analyses and *in vitro* validation highlighted *MUC16* as a markedly upregulated gene in LUAD, whose higher expression was associated with poorer patient outcomes. Functional and mechanistic experiments further suggested that *MUC16* may promote malignant phenotypes in LUAD cells, at least in part, through FAK-mediated activation of PI3K/AKT signaling.

**Conclusion:**

We developed an EGRG-based prognostic signature for LUAD, which may provide a useful reference for risk stratification and therapeutic decision-making. Further evidence suggested that *MUC16* may contribute to LUAD progression, at least in part, through FAK-mediated activation of PI3K/AKT signaling, supporting its potential value as a prognostic biomarker and a candidate for further therapeutic investigation.

## 1 Introduction

Globally, lung cancer accounted for approximately 2.5 million new cases and 1.8 million deaths in 2022, reflecting its substantial and persistent disease burden (1). Lung adenocarcinoma (LUAD) represents a major subtype of non-small cell lung cancer and continues to pose a substantial clinical challenge. While targeted therapies and immunotherapeutic strategies have markedly expanded treatment options, heterogeneous treatment responses and acquired resistance continue to limit durable clinical benefit (2, 3). Therefore, identifying key prognostic biomarkers for LUAD and elucidating their potential molecular mechanisms are important for improving patient survival.

As a pivotal intracellular organelle, the endoplasmic reticulum (ER) coordinates protein folding, post-translational modification, and trafficking. When ER homeostasis is disrupted, immature or aberrantly folded proteins may accumulate within the ER lumen, thereby activating cellular stress responses (4). If the accumulation of these abnormal proteins exceeds the processing capacity of the ER, endoplasmic reticulum stress (ER stress) will occur. Persistent ER stress has been linked to several malignant phenotypes, including immune evasion, metabolic reprogramming, and therapeutic resistance (5). In the secretory pathway, the Golgi apparatus further contributes to protein glycosylation, sorting, and packaging (6). These two organelles are functionally connected rather than isolated. Impaired Golgi function can interfere with protein processing and trafficking, leading to the retention of immature proteins in the ER and increasing the burden on its folding machinery, which may further intensify ER stress. Conversely, prolonged ER stress may disturb Golgi structure and polarity, thereby compromising Golgi function. Through this bidirectional crosstalk, a vicious cycle may develop, contributing to intracellular proteostasis disruption and malignant progression (7–9). Previous studies have constructed prognostic signatures based on genes involved in ER stress or Golgi function to predict clinical outcomes in LUAD (10, 11). Although the ER and Golgi apparatus are biologically distinct yet closely interconnected, signatures derived from only one gene category may not fully capture their combined influence on tumor progression and prognosis. Therefore, integrated analysis of endoplasmic reticulum stress- and Golgi apparatus-related genes may provide a more comprehensive framework for prognostic assessment, immune microenvironment characterization, and identification of key molecular drivers in LUAD.

We integrated endoplasmic reticulum stress- and Golgi apparatus-related genes (EGRGs) to develop a machine learning-based prognostic signature for LUAD, thereby enabling more refined prognostic stratification. Furthermore, *in vitro* functional and mechanistic experiments were performed to investigate the role of *MUC16* in LUAD progression.

## 2 Methods

### 2.1 Collection of public transcriptomic data and gene sets

Transcriptome-wide data and corresponding clinicopathological information for LUAD were accessed through TCGA (12). Two LUAD cohorts (GSE72094 and GSE30219) were obtained from GEO for model evaluation, while two additional independent cohorts (GSE26939 and GSE31210) were used for external validation of the final 17-gene prognostic signature (13–16). Pretreatment transcriptomic data from 16 patients with NSCLC treated with anti-PD-1 therapy were obtained from the GSE126044 cohort in GEO (17). Gene sets related to the Golgi apparatus and endoplasmic reticulum stress were obtained from MSigDB.

### 2.2 Differential expression and functional annotation

Using the limma R package, we screened the TCGA-LUAD transcriptomic data for genes with significant differential expression (|log_2_FC| > 2, adjusted *P* < 0.05) (18). GO, KEGG, and GSEA analyses were implemented through the “clusterProfiler” R package (19).

### 2.3 Development and evaluation of the prognostic signature and nomogram

Prognosis-related candidate genes were first screened from the endoplasmic reticulum stress- and Golgi apparatus-related differentially expressed genes by univariate Cox analysis. These genes were then entered into a machine-learning pipeline to generate prognostic models in the TCGA-LUAD cohort. Model performance was subsequently evaluated in independent GEO cohorts with Harrell’s concordance index (C-index), and the model with the highest mean C-index was chosen for further analysis. The Random Survival Forest (RSF) was ultimately retained as the optimal model. An initial RSF model was fitted using all 41 candidate genes with 1,000 trees, a node size of 4, and a random seed of 123. Variable selection was then performed using the minimal-depth method implemented in the var.select() function of the “randomForestSRC” R package, with the conservativeness level set to “high.” Genes with minimal-depth values below the data-driven threshold of 7.4904 were retained, yielding the final 17-gene signature. Variable importance was subsequently calculated to rank the retained genes. Prognostic performance was evaluated using Kaplan–Meier survival analysis and time-dependent ROC curves (20). STRING was used to analyze interactions among the 17 signature genes, with the minimum required interaction score set to 0.150. The resulting network was imported into Cytoscape for visualization and topological analysis. A nomogram based on risk score and clinical variables was generated with the “rms” R package, and calibration curves were used for validation.

### 2.4 Analysis of tumor microenvironment immune characteristics

EPIC, MCP-counter, TIMER, and CIBERSORT were applied to profile immune infiltration. Spearman correlation was then used to link immune cell abundance with the risk score and signature genes. The ESTIMATE algorithm generated stromal, immune, and ESTIMATE scores to characterize the tumor microenvironment. TIDE was applied to estimate immune escape potential, and the OCLR-derived RNAss was used to reflect stemness features (21–23).

### 2.5 Characterization of genomic alterations and TMB

For each sample in TCGA-LUAD, tumor mutational burden (TMB) was estimated from the corresponding mutation annotation file. The overall mutational landscape, including frequently altered genes, was summarized and visualized using the maftools R package (24).

### 2.6 Drug sensitivity analysis

Using the oncoPredict R package (25), transcriptomic profiles from TCGA-LUAD were integrated with GDSC drug response data to infer the response of each sample to anticancer agents. Predicted IC50 values were calculated for each sample, and their relationships with the risk score, together with differences across risk subgroups, were subsequently analyzed.

### 2.7 Cell culture, siRNA-mediated knockdown, and *MUC16* overexpression

Servicebio (Wuhan, China) supplied the LUAD cell lines PC9 and H1975, as well as the normal bronchial epithelial cell line BEAS-2B. Cells were maintained in RPMI-1640 (Beyotime, C2721) supplemented with 10% fetal bovine serum (Beyotime, C0226S) at 37°C in a humidified atmosphere containing 5% CO_2_. *MUC16*-targeting siRNAs were purchased from MedChemExpress and transfected into cells using SweTransRNA Transfection Reagent (Servicebio, G1806) according to the manufacturer’s instructions. The siRNA sequences targeting *MUC16* were provided in **Supplementary Table 1**. The plasmid used for *MUC16* overexpression was synthesized and constructed by General Biosystems (Anhui, China). It encoded the C-terminal region of *MUC16*, including the membrane-proximal extracellular region, transmembrane domain, and cytoplasmic tail.

### 2.8 Evaluation of cell growth and DNA synthesis

For CCK-8 assays, cells were seeded in 96-well plates at a density of 1000 cells per well, and cell viability was assessed using a CCK-8 kit (Beyotime, C0037) at 0, 24, 48, and 72 h. At each time point, 10 μL of CCK-8 reagent was added to each well, followed by incubation at 37°C for 2 h, and absorbance was measured at 450 nm. Similar colorimetric assays have been used to evaluate cell viability and proliferation in previous studies (26, 27). For EdU staining, EdU incorporation was detected using the EdU kit (Beyotime, C0078S) according to the manufacturer’s instructions, followed by nuclear counterstaining with Hoechst 33342. Proliferative activity was quantified according to EdU incorporation.

### 2.9 Assessment of cell migration and invasion

Transwell chambers with 8-μm pores (Beyotime, FTW164) were used to evaluate cell migration and invasion, with Matrigel (Beyotime, C0372) coating applied only in the invasion assay. After 24 h, cells that had migrated or invaded to the lower membrane surface were fixed, stained with crystal violet, and imaged. For each assay, 3 × 10^^4^ cells were seeded into the upper chamber.

### 2.10 Apoptosis assay

At 48 h post-transfection, floating and adherent cells from each treatment group were harvested. Adherent cells were collected by trypsinization and pooled with the floating fraction. Following two washes with ice-cold PBS, cells were resuspended in binding buffer, stained with EGFP Annexin V/PI (Servicebio, G1510) for 25 min in the dark, and apoptotic cells were quantified using a NovoCyte 2000R flow cytometer.

### 2.11 Protein expression analysis

RIPA buffer (Beyotime, P0013B) containing PMSF and protease/phosphatase inhibitor cocktails was used for total protein extraction. Protein levels were measured using the bicinchoninic acid (BCA) assay. Equivalent protein samples were then separated on 10% SDS–polyacrylamide gels, followed by blotting onto PVDF membranes (Immobilon-P, ISEQ00010). After 5% BSA blocking, primary antibody incubation was carried out overnight at 4°C for MUC16 (Proteintech, 20077-1-AP), β-Tubulin (Proteintech, 66240-1-Ig), p-PI3K (MCE, HY-P80846), t-PI3K (MCE, HY-P80867), p-AKT (MCE, HY-P80789), and t-AKT (MCE, HY-P85759). After primary antibody incubation, membranes were exposed to HRP-linked goat anti-rabbit IgG (Proteintech, RGAR001) or goat anti-mouse IgG (Proteintech, RGAM001) for 1 h under ambient conditions. Protein signals were detected using the Omni-ECL™ Basic Chemiluminescence Detection Kit (Epizyme, SQ202). For PI3K/AKT pathway inhibition experiments, the PI3K inhibitor Buparlisib (MCE, HY-70063) was added to MUC16-overexpressing PC9 and H1975 cells for 24 h before protein extraction. Based on the IC50 values determined by CCK-8 assays, Buparlisib (BKM120) was used at one-quarter of the corresponding IC50 concentration for each cell line. This concentration was selected to minimize nonspecific effects on cell viability while maintaining sufficient inhibition of PI3K/AKT signaling.

### 2.12 Co-Immunoprecipitation assay

Total protein from PC9 cells was extracted using mild lysis buffer for Western blotting and immunoprecipitation supplemented with protease inhibitors. Reciprocal co-immunoprecipitation was performed using anti-MUC16 or anti-FAK (MCE, HY-P80125) antibodies, with the corresponding species-matched IgG as a negative control. The immune complexes were captured using Protein A/G agarose beads (Beyotime, P2055) and subsequently analyzed by Western blotting for MUC16 and FAK.

### 2.13 Statistical methods

R (version 4.4.3) was used as the analytical platform for bioinformatic analyses, and experimental data were analyzed using GraphPad Prism (version 10.1.2). At least three biological replicates were included for all experiments. Comparisons among groups were performed using one-way or two-way analysis of variance, depending on the study design. Survival was analyzed with the Kaplan–Meier method and compared by the log-rank test. Correlations were assessed using Spearman’s correlation analysis. Statistical significance was defined as *P* < 0.05 (*P < 0.05, **P < 0.01, ***P < 0.001).

## 3 Results

### 3.1 Functional landscape of EGR-DEGs in LUAD

**Figure 1:**
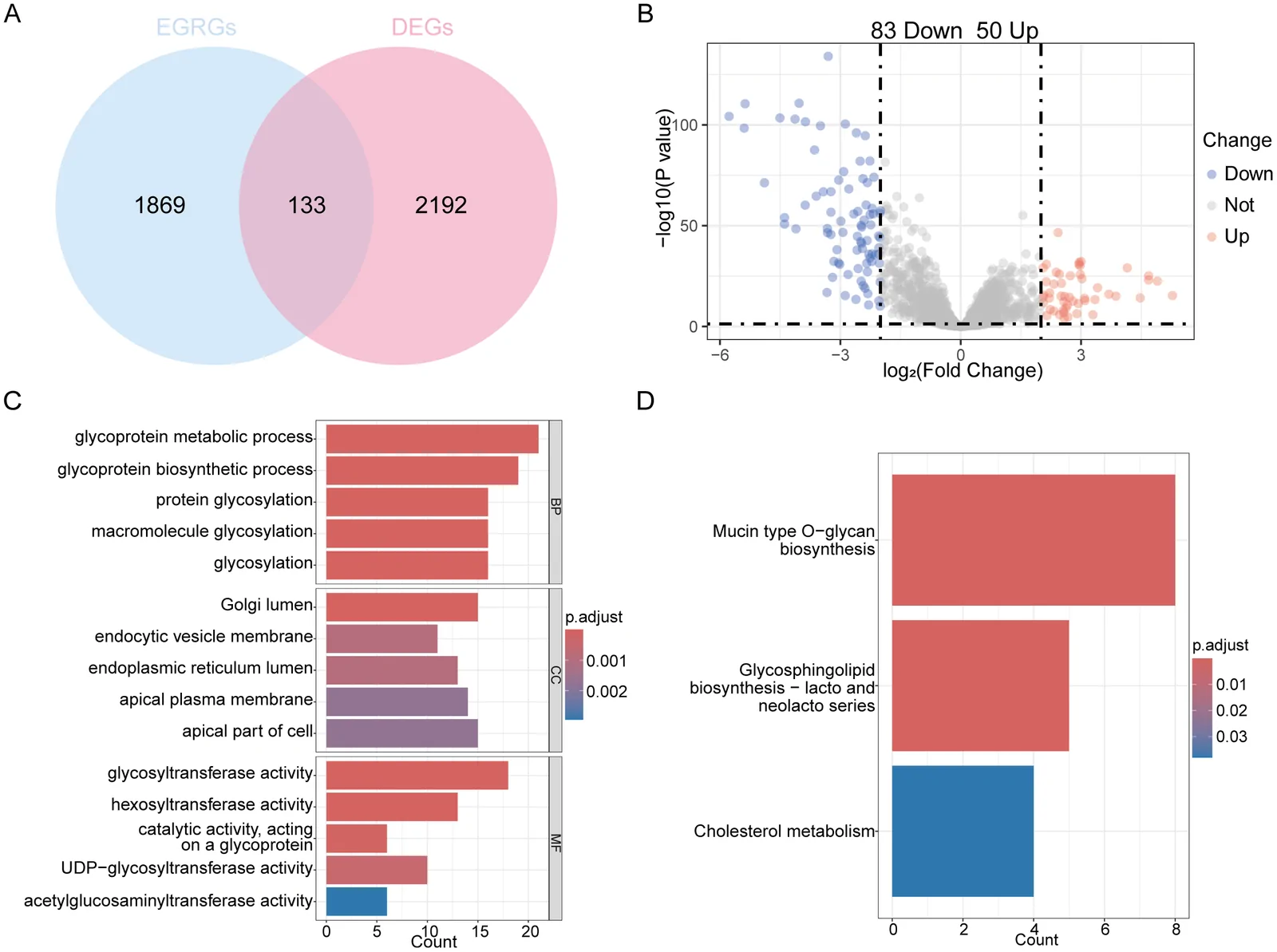
Screening and functional annotation of EGR-DEGs in LUAD. **(A)** Overlap between EGRGs and DEGs, yielding 133 EGR-DEGs. **(B)** Volcano plot of the 133 EGR-DEGs. **(C)** GO enrichment of EGR-DEGs. **(D)** KEGG pathway enrichment of EGR-DEGs.

Comparison between LUAD tissues and normal lung tissues identified 2,325 differentially expressed genes (DEGs). Intersecting these DEGs with the 2,002 endoplasmic reticulum stress- and Golgi apparatus-related genes (EGRGs) yielded 133 endoplasmic reticulum stress- and Golgi apparatus-related DEGs (EGR-DEGs) (**Figure 1A**). Their differential expression distribution was illustrated by the volcano plot (**Figure 1B**). GO enrichment analysis identified glycoprotein metabolic process, Golgi lumen, and glycosyltransferase activity as the major enriched functional categories (**Figure 1C**). KEGG analysis further revealed significant enrichment of the mucin-type O-glycan biosynthesis pathway (**Figure 1D**). GSEA showed that ribosome biogenesis, rRNA processing, amino acid and monosaccharide metabolic processes, and DNA repair were significantly activated in LUAD (**Supplementary Figure 1A**). By contrast, positive modulation of cell adhesion, endocytosis, leukocyte adhesion, and control of cell activation were significantly suppressed (**Supplementary Figure 1B**).

### 3.2 Construction of the prognostic model

**Figure 2:**
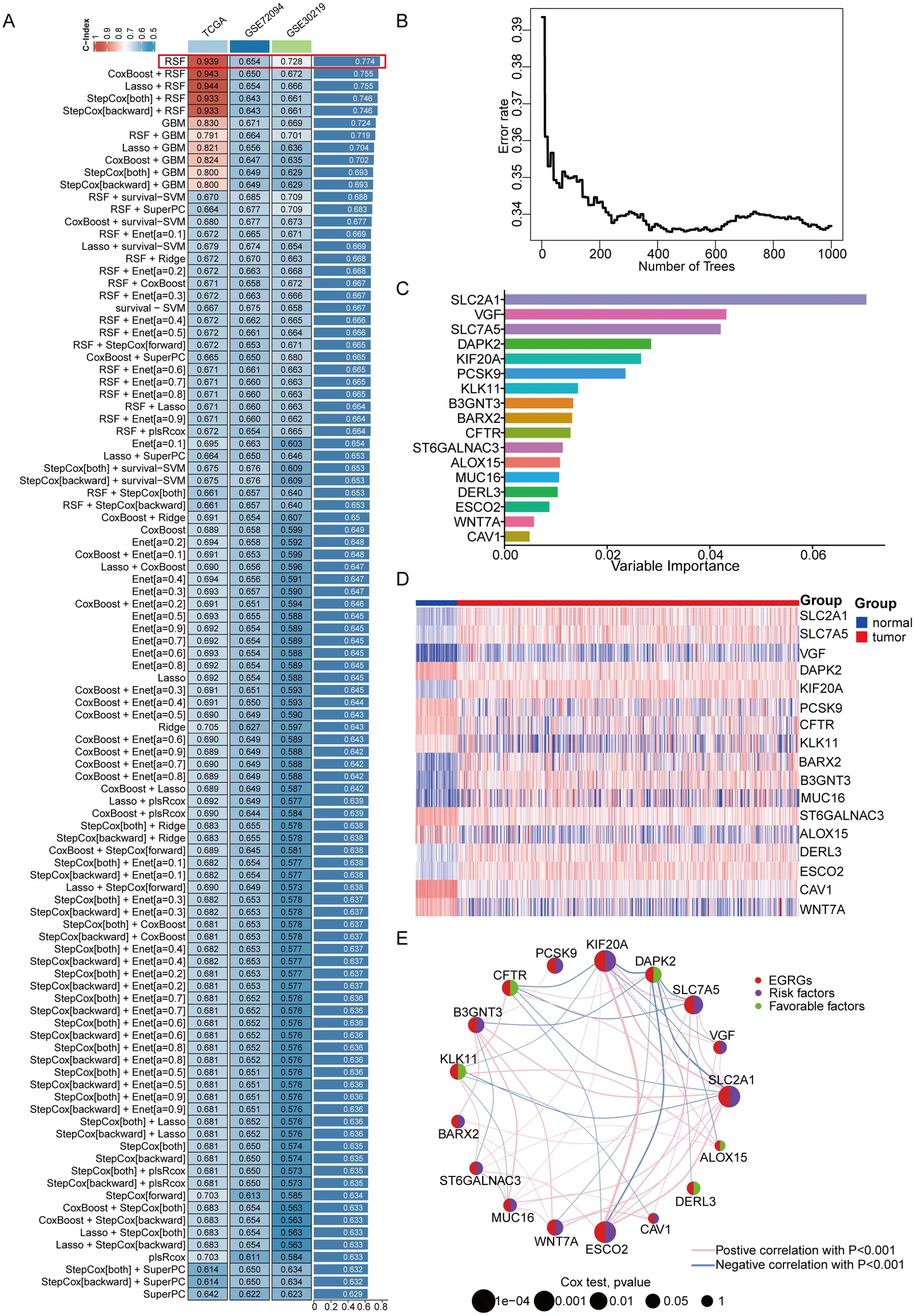
Construction of the prognostic signature. **(A)** Comparison of 101 machine learning algorithm combinations across cohorts using the C-index. **(B)** Error rate of the Random Survival Forest (RSF) model according to tree number. **(C)** Variable importance of the 17 signature genes in the RSF model. **(D)** Heatmap of signature gene expression across LUAD and normal tissues. **(E)** Correlation network of the 17 model genes, showing risk factors, favorable factors, and pairwise associations.

Of the 133 EGR-DEGs identified between tumor and normal tissues, 41 were significantly linked to patient survival. Using these candidate genes, 101 prognostic models were established, and their performance in the training and validation cohorts was compared in **Figure 2A**, with the exact C-index values provided in **Supplementary Table 2**. Among all candidate models, the Random Survival Forest achieved the highest mean C-index of 0.774 and was retained as the final model (**Figure 2A**; **Supplementary Table 2**). Minimal-depth variable selection retained 17 genes with values below the data-driven threshold of 7.4904. As the number of trees increased, the model error gradually declined and eventually became stable (**Figure 2B**), and the variable importance ranking of the 17 signature genes was shown in **Figure 2C**. The heatmap illustrated their expression profiles in LUAD and normal lung samples (**Figure 2D**). In addition, the associations between signature gene expression and prognostic factors were shown in the prognostic network plot (**Figure 2E**).

### 3.3 Validation of the prognostic signature

**Figure 3:**
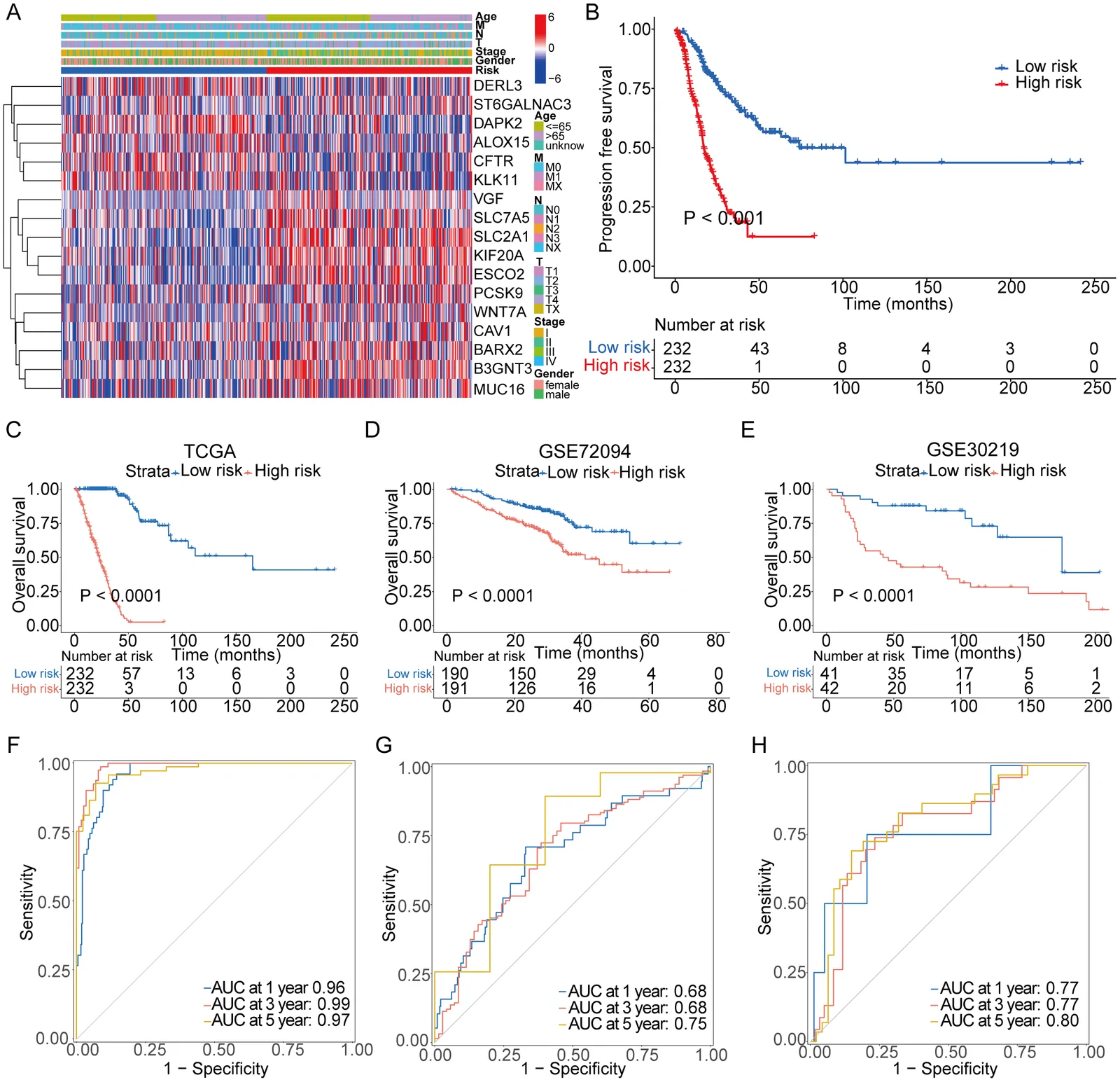
Validation of the prognostic signature in TCGA-LUAD, GSE72094, and GSE30219. **(A)** Heatmap of the 17 model genes and clinicopathological features. **(B)** Progression-free survival in the training cohort. **(C–E)** Kaplan–Meier analysis of overall survival in the three cohorts. **(F–H)** Time-dependent ROC analyses in the three cohorts.

The heatmap integrating signature gene expression with clinical variables reflected the relationships among clinical characteristics, model genes, and risk groups in the TCGA cohort (**Figure 3A**). Patients were stratified into high- and low-risk groups according to the median risk score. Patients with higher risk scores showed worse progression-free survival and overall survival than those with lower risk scores (**Figures 3B, C**). The combined risk plot provided an overview of risk-score ranking, survival outcomes, and signature gene expression in the two risk groups (**Supplementary Figure 2A**). The time-dependent ROC curves indicated strong predictive performance of the signature-derived risk score, with 1-, 3-, and 5-year AUCs of 0.96, 0.99, and 0.97, respectively (**Figure 3F**). In the two external validation cohorts, clear differences in survival outcomes and signature gene expression remained evident between the two risk groups (**Supplementary Figures 2B, C**). Kaplan–Meier analysis likewise indicated that patients in the low-risk group had more favorable overall survival (**Figures 3D, E**). The signature-derived risk score also maintained satisfactory discrimination in both validation cohorts, with all AUCs remaining above 0.65 (**Figures 3G, H**). Further validation in two additional independent cohorts, GSE26939 and GSE31210, showed that risk-score-based stratification significantly distinguished overall survival (*P* = 0.042 and *P* = 0.015, respectively). The 1-, 3-, and 5-year AUCs were 0.69, 0.69, and 0.59 in GSE26939, and 0.56, 0.61, and 0.66 in GSE31210, respectively (**Supplementary Figure 3**).

Subgroup analysis of clinical characteristics demonstrated that the risk score effectively stratified patient prognosis across subgroups defined by age, sex, TNM stage, and clinical stage (**Supplementary Figures 4A–L**). In addition, sex, TNM stage, and overall clinical stage differed noticeably across the two risk groups, whereas age showed a similar distribution (**Supplementary Figures 4M–R**).

### 3.4 Nomogram performance and validation

**Figure 4:**
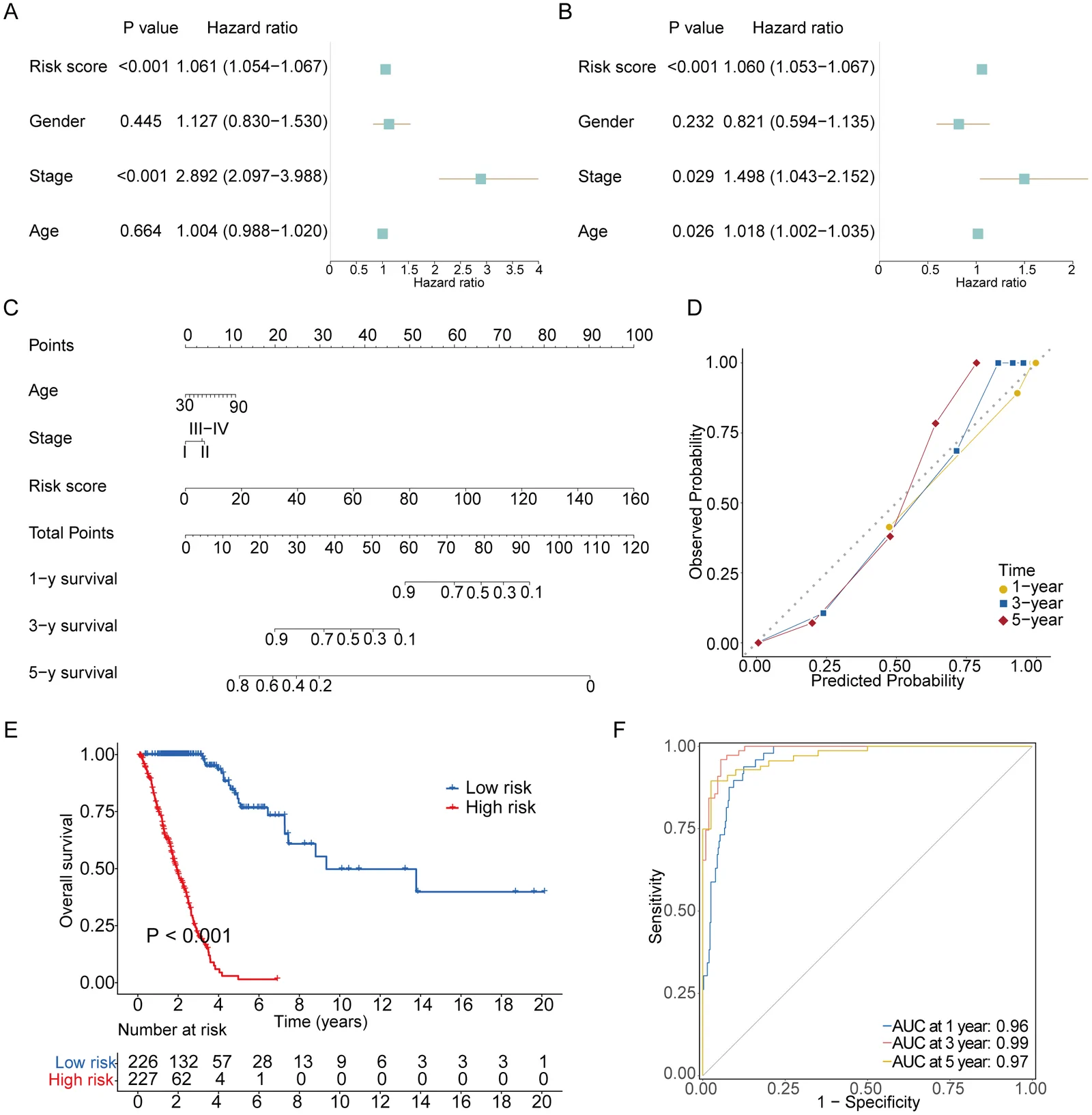
Nomogram-based survival prediction in LUAD. **(A, B)** Univariate and multivariate Cox analyses. **(C)** Nomogram for predicting 1-, 3-, and 5-year survival. **(D)** Calibration curves. **(E)** Kaplan–Meier survival analysis according to the nomogram score. **(F)** Time-dependent ROC analysis.

Univariate Cox regression identified both clinical stage and risk score as variables related to overall survival, while multivariate analysis confirmed risk score as an independent prognostic factor (*P* < 0.05; **Figures 4A, B**). **Figure 4C** shows a nomogram integrating the risk score with clinical variables for prognostic assessment in LUAD. Calibration analysis showed good agreement between predicted and observed 1-, 3-, and 5-year OS probabilities (**Figure 4D**). Stratification based on the nomogram score separated patients with distinct survival outcomes, and those with higher scores had poorer overall survival (**Figure 4E**). Likewise, the nomogram showed strong predictive performance, with AUC values above 0.9 at all evaluated time points (**Figure 4F**).

### 3.5 Immune features within the tumor microenvironment

**Figure 5:**
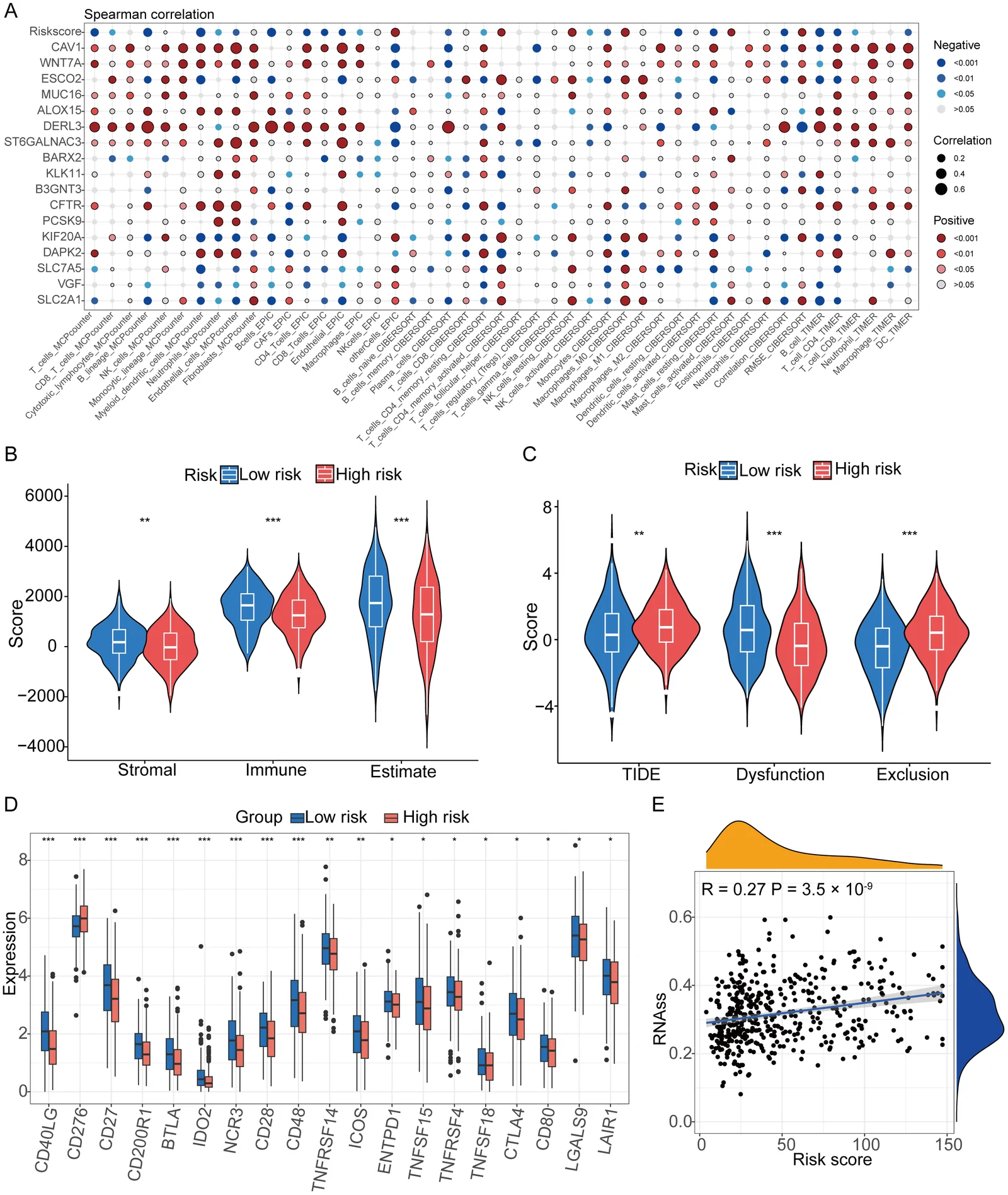
Immune microenvironment characteristics across risk subgroups. **(A)** Correlation map of immune infiltration, signature genes, and risk score. **(B)** Stromal, immune, and ESTIMATE scores across the two risk subgroups. **(C)** TIDE, dysfunction, and exclusion scores across the two risk subgroups. **(D)** Immune checkpoint gene expression patterns in low- and high-risk patients. **(E)** Correlation between risk score and RNAss. *P < 0.05; **P < 0.01; ***P < 0.001.

We next examined immune differences between the two risk groups by integrating several computational approaches to characterize the immune microenvironment in the TCGA-LUAD cohort. Higher risk scores were accompanied by reduced infiltration of several immune cell populations, including CD8^+^ T cells, CD4^+^ T cells, and myeloid dendritic cells (**Figure 5A**). Patients in the low-risk subgroup displayed increased stromal, immune, and ESTIMATE scores relative to those in the high-risk group (**Figure 5B**). In contrast, the high-risk group showed a more pronounced immune-evasive profile, characterized by higher TIDE scores (**Figure 5C**). Higher CD276 expression was observed in the high-risk subgroup (**Figure 5D**), which also showed enhanced stemness features (**Figure 5E**).

To further explore the clinical relevance of the signature in immunotherapy, the established RSF model was applied to the GSE126044 cohort of patients with NSCLC treated with anti-PD-1 therapy. Non-responders tended to have higher RSF-predicted risk scores than responders, although the difference did not reach statistical significance (*P* = 0.141; **Supplementary Figure 5**).

### 3.6 Somatic mutation features and TMB across risk groups

The high-risk subgroup displayed a greater mutational burden than the low-risk subgroup (**Supplementary Figure 6A**), and the risk score showed a positive correlation with TMB (**Supplementary Figure 6B**). When risk score and TMB were jointly considered, patients with low risk and high TMB showed the most favorable survival outcome (**Supplementary Figure 6C**). In addition, although the major mutated genes were similar across groups, the overall mutation burden remained more pronounced in the high-risk subgroup (**Supplementary Figures 6D, E**).

### 3.7 Predicted therapeutic response across risk strata

**Figure 6:**
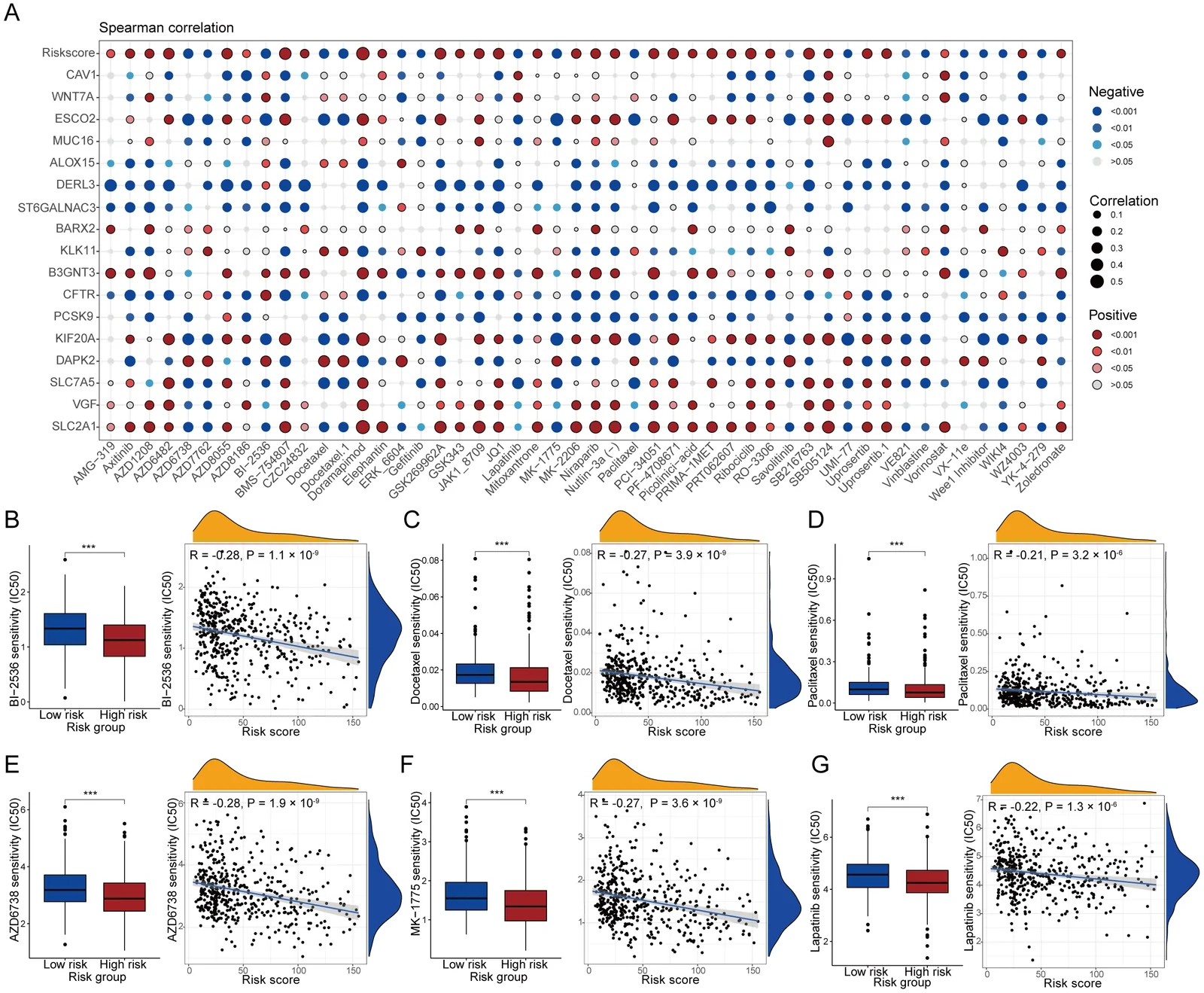
Drug sensitivity analysis. **(A)** Correlations of risk score and signature gene expression with drug IC_50_ values. **(B–G)** Predicted IC_50_ values of BI-2536, Docetaxel, Paclitaxel, MK-1775, AZD6738, and lapatinib in the two risk groups, along with their associations with risk score. ***P < 0.001.

Predicted IC_50_ values were estimated for each drug in TCGA-LUAD samples. The risk score was significantly correlated with the predicted sensitivity to multiple drugs (**Figure 6A**). Overall, higher risk scores were associated with higher predicted IC_50_ values for most agents, suggesting greater predicted drug sensitivity in the low-risk group. However, several agents exhibited the opposite pattern, with significantly lower predicted IC_50_ values in the high-risk group (**Figures 6B–G**). Notably, the clinically relevant agents docetaxel and paclitaxel showed lower predicted IC50 values in the high-risk group and were negatively correlated with the risk score (docetaxel: *R* = −0.27, *P* = 3.9 × 10^-9^; paclitaxel: *R* = −0.21, *P* = 3.2 × 10^-6^). Similar patterns were observed for BI-2536, AZD6738, MK-1775, and lapatinib.

### 3.8 *MUC16* and its prognostic significance in LUAD

**Figure 7:**
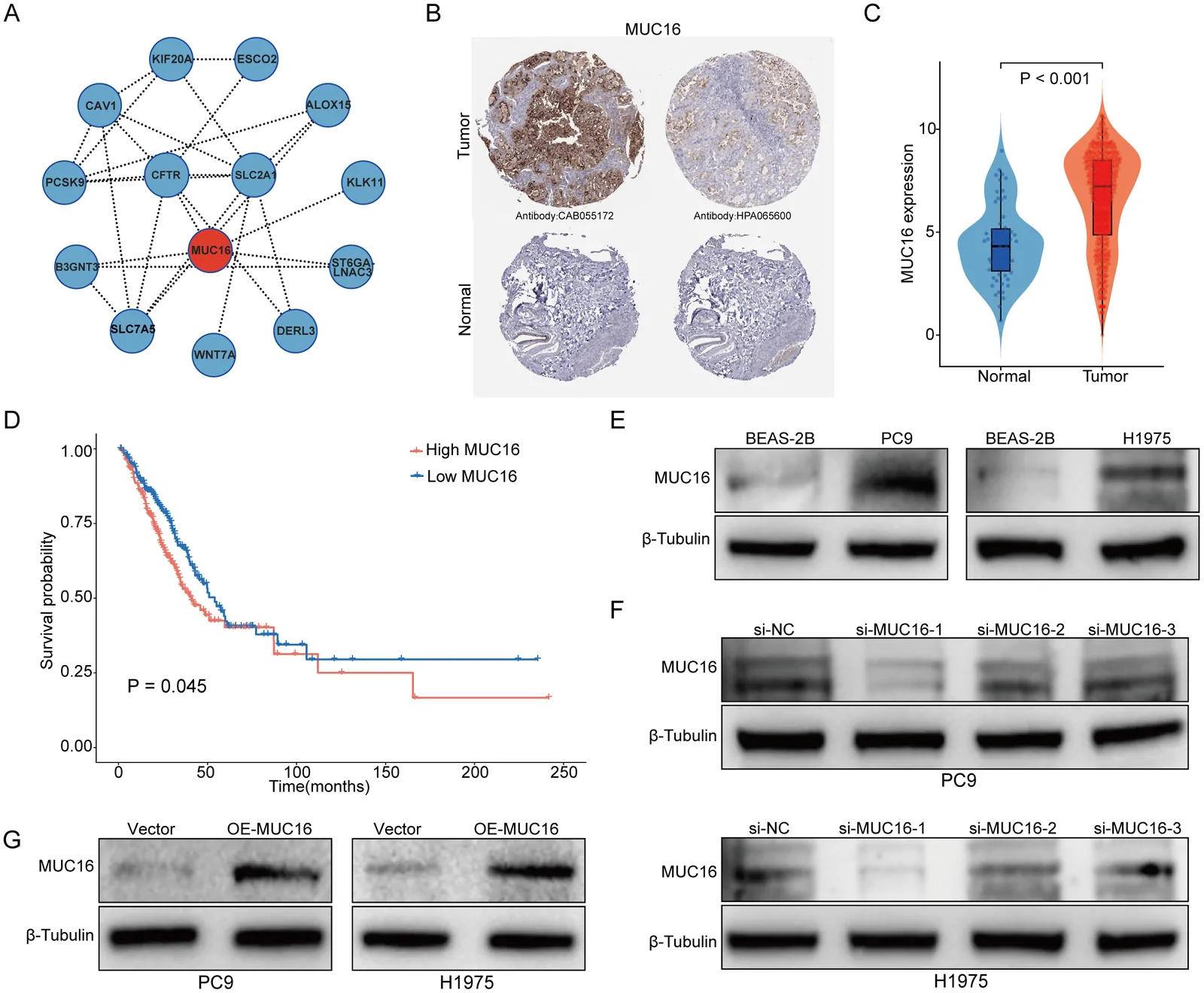
Identification and validation of *MUC16* as a pivotal prognostic biomarker in LUAD. **(A)** PPI network of the signature genes. **(B)** Human Protein Atlas images showing MUC16 staining in normal and tumor samples. Reproduced under the Creative Commons Attribution 4.0 International (CC BY 4.0) license. Image credit: Human Protein Atlas. **(C)** *MUC16* expression in normal and LUAD tissues. **(D)** Survival stratification by *MUC16* expression in the TCGA-LUAD cohort. **(E)** Immunoblot assessment of basal MUC16 levels across BEAS-2B, PC9, and H1975 cells. **(F)** Western blot assessment of *MUC16* knockdown efficiency mediated by three independent small interfering RNAs (si-MUC16-1/2/3) in PC9 and H1975 cells. **(G)** Immunoblot confirmation of *MUC16* overexpression in the two LUAD cell models.

The interactions among the 17 signature genes were analyzed using protein–protein interaction (PPI) analysis. The results showed that, except for *VGF*, *BARX2*, and *DAPK2*, the remaining 14 genes exhibited interactions (**Supplementary Figure 7**). Betweenness centrality analysis was further performed using Cytoscape to score each gene and to visualize the PPI network (**Figure 7A**), in which *CFTR*, *SLC2A1*, and *MUC16* ranked as the top three genes. Comparative analyses showed that *SLC2A1* had the strongest association with overall survival in the univariate Cox analysis and the highest positive correlation with the risk score, whereas *MUC16* also showed a significant adverse prognostic association and a positive correlation with the risk score. In contrast, *CFTR* was associated with favorable overall survival and negatively correlated with the risk score (**Supplementary Table 3**). Although *MUC16* did not exhibit superior statistical performance, it was prioritized based on the combined evidence of network centrality, prognostic relevance, positive association with the risk score, and its close biological relevance to ER- and Golgi-dependent glycoprotein processing. Immunohistochemical data from the Human Protein Atlas database showed that MUC16 protein expression was higher in LUAD compared with normal lung tissues (**Figure 7B**). Consistent results were observed in the TCGA-LUAD cohort, where tumor samples exhibited significantly higher *MUC16* expression than normal tissues (**Figure 7C**), and elevated *MUC16* expression was associated with unfavorable survival outcomes, further supporting its prognostic relevance in LUAD (**Figure 7D**).

Basal MUC16 protein expression was compared among BEAS-2B, PC9, and H1975 cells by immunoblotting. MUC16 levels were markedly higher in PC9 and H1975 cells than in BEAS-2B cells (**Figure 7E**). Subsequently, *MUC16* knockdown models were established in both LUAD cell lines, and Western blotting confirmed that si-*MUC16–*1 efficiently reduced MUC16 protein expression (**Figure 7F**). Therefore, si-*MUC16–*1 was selected for subsequent functional assays and mechanistic validation. In addition, a plasmid encoding the C-terminal region of MUC16 (MUC16-C), including the membrane-proximal extracellular region, transmembrane domain, and cytoplasmic tail, was transfected into both LUAD cell lines, and successful MUC16-C overexpression was confirmed (**Figure 7G**). In the subsequent experiments, *MUC16* overexpression refers to overexpression of this MUC16-C construct.

### 3.9 *MUC16* promotes the malignant phenotype of lung adenocarcinoma cells

**Figure 8:**
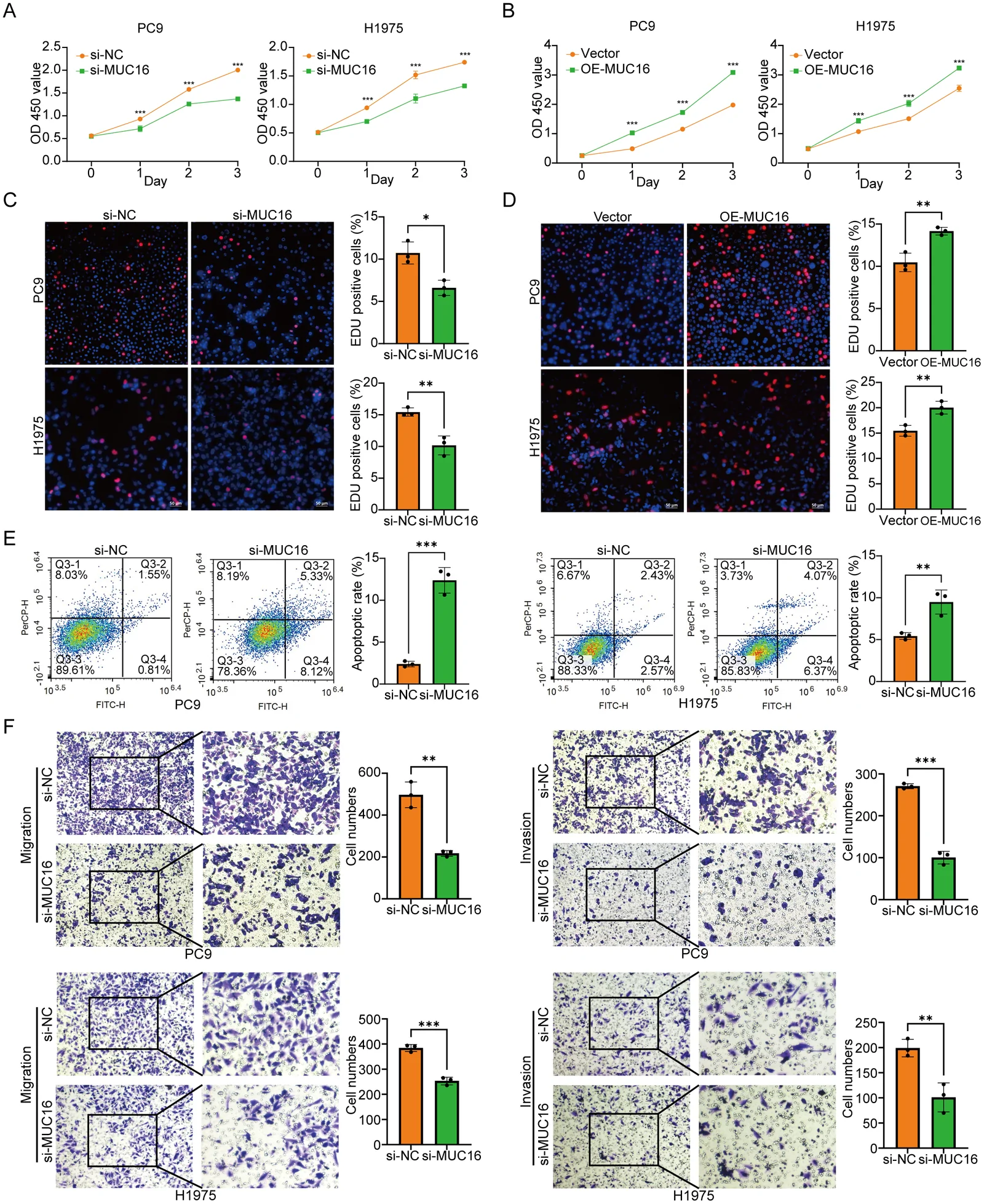
Effects of *MUC16* on LUAD cell malignant phenotypes. **(A, B)** CCK-8 assays after *MUC16* knockdown or *MUC16* overexpression. **(C, D)** EdU staining and quantification of EdU-positive cells. **(E)** Flow cytometric analysis of apoptosis after *MUC16* knockdown. **(F)** Transwell migration and invasion assays after *MUC16* knockdown. Data are presented as the mean ± SD from three independent experiments. *P < 0.05; **P < 0.01; ***P < 0.001.

*MUC16* knockdown significantly suppressed proliferation in PC9 and H1975 cells, as shown by reduced CCK-8 absorbance and lower proportions of EdU-positive cells (**Figures 8A, C**). In contrast, *MUC16* overexpression markedly increased proliferative capacity and DNA synthesis activity in these cells (**Figures 8B, D**). Flow cytometry showed an increased apoptotic fraction after *MUC16* depletion in both LUAD cell lines (**Figure 8E**). Consistently, fewer cells passed through the Transwell membrane after *MUC16* knockdown, indicating reduced migratory and invasive potential (**Figure 8F**). These results suggest that *MUC16* may promote malignant phenotypes in LUAD cells.

### 3.10 *MUC16* activates PI3K/AKT signaling in LUAD cells

**Figure 9:**
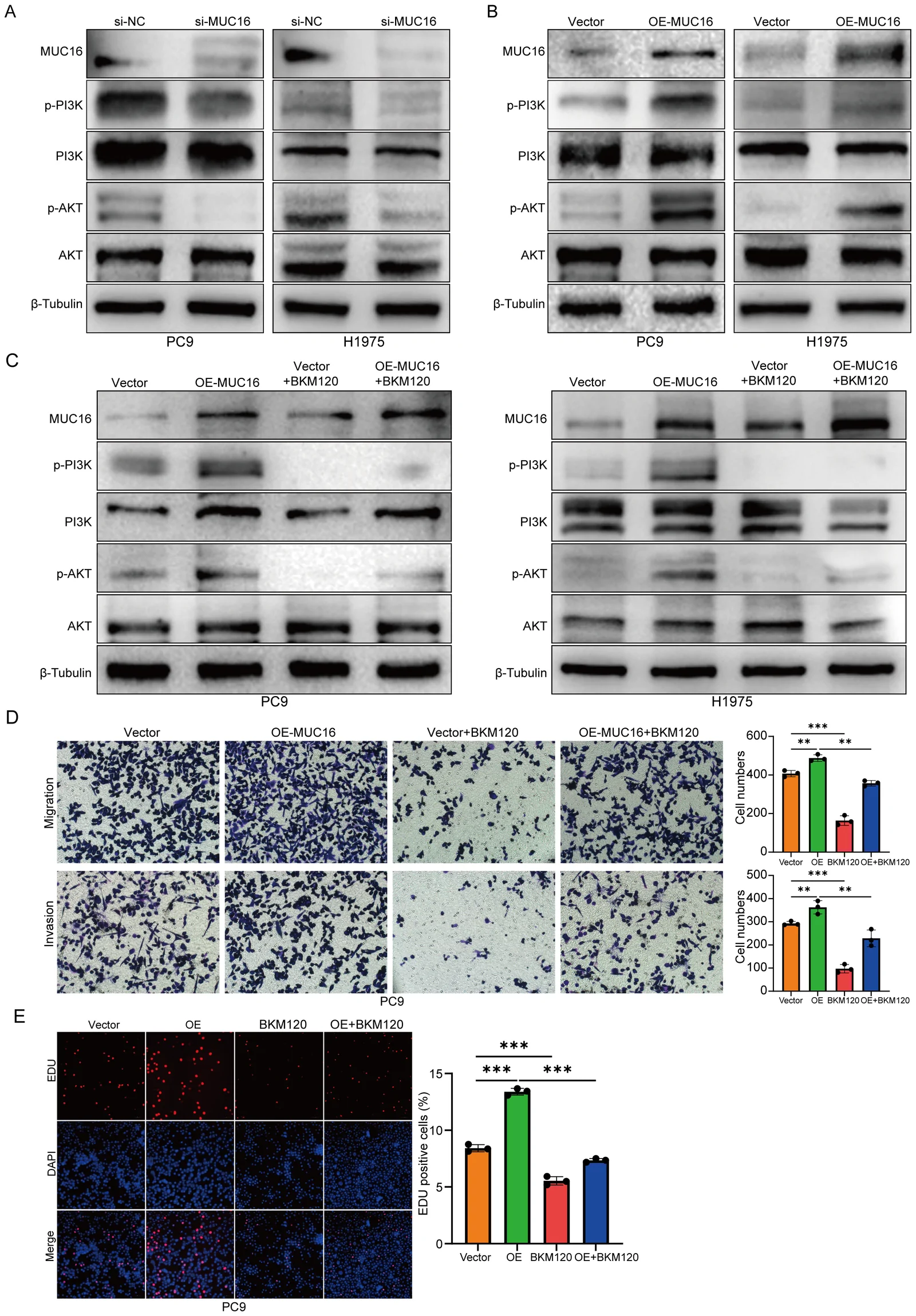
MUC16 influences the PI3K/AKT pathway. **(A, B)** Immunoblot assessment of MUC16 and PI3K/AKT signaling activity following *MUC16* knockdown or overexpression. **(C)** Effect of Buparlisib (BKM120) on MUC16-induced PI3K/AKT activation. **(D)** Effects of Buparlisib on the migration and invasion of MUC16-overexpressing PC9 cells, as assessed by Transwell assays. **(E)** Effects of Buparlisib on the proliferation of MUC16-overexpressing PC9 cells, as assessed by EdU incorporation assays. Data are presented as the mean ± SD from three independent experiments. **P < 0.01, ***P < 0.001.

Single-gene GSEA of the TCGA-LUAD cohort showed enrichment of PI3K/AKT-related pathways among genes linked to high MUC16 expression in both GO and Hallmark annotations. These findings suggest that *MUC16* may be closely linked to the activation of the PI3K/AKT signaling axis (**Supplementary Figure 8**). To further support this inference, we assessed pathway-related proteins by Western blot. In both PC9 and H1975 cells, depletion of *MUC16* reduced p-PI3K and p-AKT levels, while *MUC16* overexpression produced the opposite effect (**Figures 9A, B**).

To further determine whether *MUC16* exerts its effects through PI3K/AKT signaling, *MUC16*-overexpressing cells were treated with Buparlisib (BKM120), a PI3K inhibitor. According to the CCK-8-derived IC50 values, Buparlisib was administered for 24 h at one-quarter of the corresponding IC50 concentration: 0.67 μM in PC9 cells and 0.40 μM in H1975 cells (**Supplementary Figures 9A, B**). Western blot analysis showed that Buparlisib (BKM120) effectively reversed the *MUC16* overexpression-induced increases in p-PI3K and p-AKT levels (**Figure 9C**). In PC9 cells, Buparlisib (BKM120) also significantly reduced the enhanced migration, invasion, and EdU incorporation induced by MUC16 overexpression (**Figures 9D, E**). These findings suggest that *MUC16* may enhance the malignant biological behavior of LUAD cells through activation of PI3K/AKT signaling.

### 3.11 FAK mediates MUC16-induced PI3K/AKT activation

**Figure 10:**
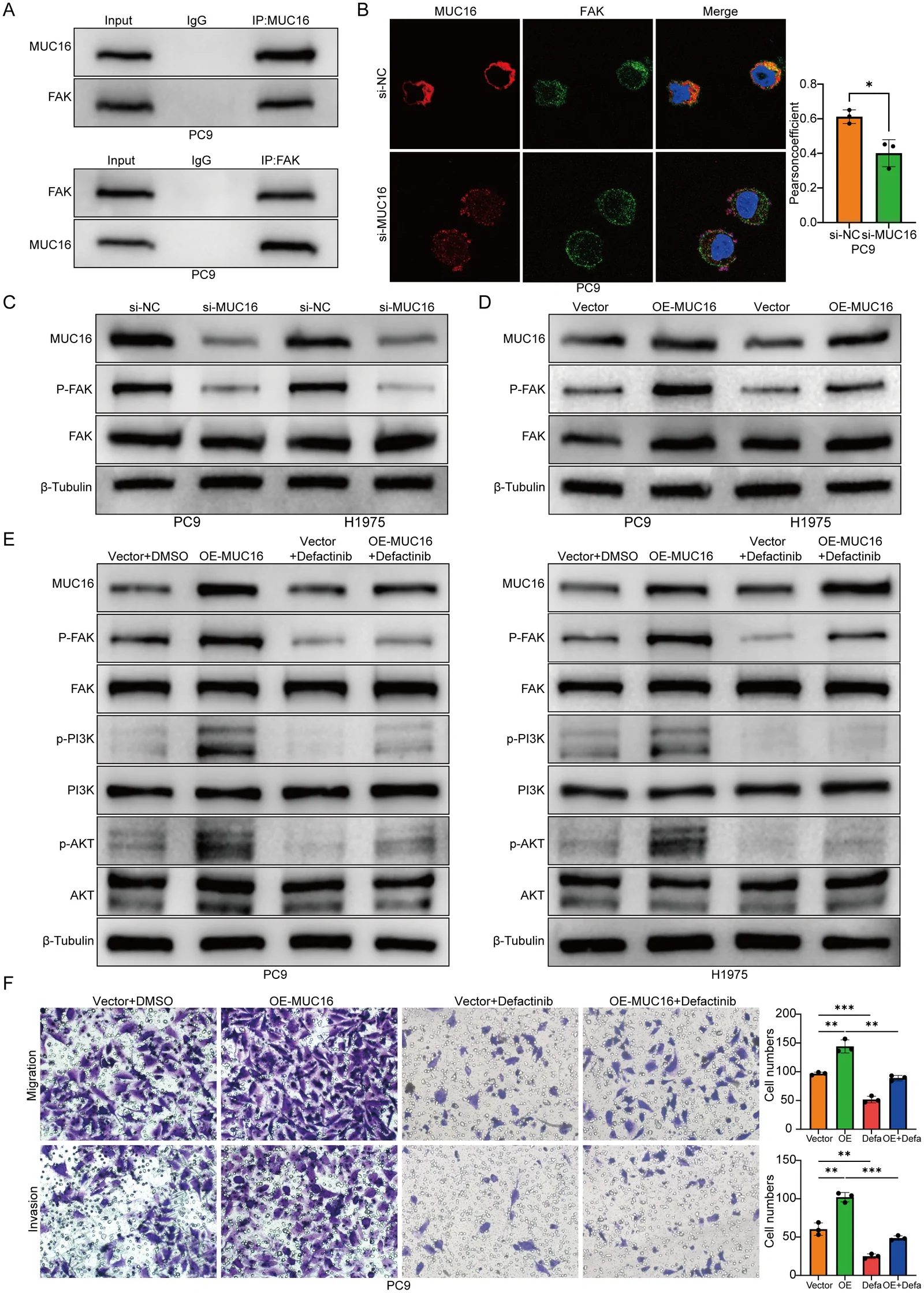
MUC16 activates PI3K/AKT signaling through FAK. **(A)** Reciprocal Co-IP analysis of the MUC16–FAK association in PC9 cells. **(B)** Immunofluorescence colocalization of MUC16 and FAK in PC9 cells following *MUC16* silencing. **(C, D)** Western blot analysis of FAK phosphorylation after *MUC16* knockdown or overexpression in PC9 and H1975 cells. **(E)** Effects of the FAK inhibitor defactinib on FAK/PI3K/AKT signaling in MUC16-overexpressing cells. **(F)** Transwell migration and invasion assays in PC9 cells. Data are presented as the mean ± SD from three independent experiments. *P < 0.05, **P < 0.01, ***P < 0.001.

Previous evidence from pancreatic ductal adenocarcinoma has shown that MUC16 associates with FAK and is linked to downstream AKT signaling (28). We therefore examined whether FAK serves as an intermediate signaling node linking MUC16 to PI3K/AKT activation in LUAD. Reciprocal Co-IP assays showed that MUC16 and FAK were present in the same protein complex in PC9 cells (**Figure 10A**). Immunofluorescence analysis further revealed spatial colocalization between MUC16 and FAK, which was reduced following MUC16 silencing (**Figure 10B**). In both PC9 and H1975 cells, MUC16 knockdown decreased FAK phosphorylation, whereas MUC16 overexpression increased FAK phosphorylation (**Figures 10C, D**). In both PC9 and H1975 cells, defactinib markedly attenuated the increase in FAK phosphorylation induced by MUC16 overexpression, accompanied by corresponding reductions in PI3K and AKT phosphorylation (**Figure 10E**). In PC9 cells, defactinib significantly attenuated the enhanced migration and invasion induced by MUC16 overexpression (**Figure 10F**).

## 4 Discussion

Lung cancer continues to impose a substantial global mortality burden (1). LUAD is characterized by pronounced molecular heterogeneity, while the frequent emergence of resistance to EGFR-TKIs continues to limit therapeutic efficacy (2, 3). The endoplasmic reticulum and Golgi apparatus play central roles in maintaining intracellular proteostasis, and dysfunction of these organelles is closely linked to malignant progression. In this study, we constructed a machine learning-based prognostic signature for LUAD using endoplasmic reticulum stress- and Golgi apparatus-related genes and further identified *MUC16* as a key gene. Subsequent analyses and *in vitro* experiments suggested that MUC16 may contribute to LUAD progression, at least in part, by activating PI3K/AKT signaling through FAK.

We systematically analyzed the characteristics of EGRGs and examined their associations with patient outcomes and the immune microenvironment. By screening differentially expressed EGRGs, we constructed a 17-gene prognostic signature using a Random Survival Forest approach. This signature divided patients into distinct prognostic subgroups, with higher risk scores associated with poorer survival outcomes, including shorter OS and PFS. Multivariable Cox regression further showed that the risk score served as an independent prognostic factor in LUAD, and ROC analysis supported the stable prognostic performance of the signature-derived risk score. Further validation in the independent GSE26939 and GSE31210 cohorts provided additional support for the prognostic stratification ability of the 17-gene signature. In addition, immune infiltration analysis indicated that patients with higher risk scores had lower levels of antitumor immune cell populations, including CD8^+^ T cells, CD4^+^ T cells, and myeloid dendritic cells, suggesting that endoplasmic reticulum stress and Golgi dysfunction may contribute to immune evasion by remodeling the tumor microenvironment. In the exploratory analysis of the GSE126044 anti-PD-1-treated NSCLC cohort, although the difference did not reach statistical significance, non-responders tended to have higher RSF-predicted risk scores than responders, providing preliminary support for a potential association between higher model-predicted risk and poorer immunotherapy response. However, in the immune infiltration analysis, we found that *MUC16* expression did not show consistent negative correlations with these immune cell populations. This observation suggests that the immune infiltration pattern associated with the risk score may reflect the combined contribution of the 17-gene signature rather than the effect of *MUC16* alone.

*MUC16* was retained as a core gene in the prognostic signature, suggesting its potential functional importance in LUAD progression. Notably, as the largest known membrane-bound mucin, *MUC16* is highly dependent on endoplasmic reticulum and Golgi apparatus homeostasis for its maturation (29). Previous studies have linked *MUC16* to multiple cancers, such as pancreatic, ovarian, and breast malignancies (30–32), whereas its role in LUAD remains incompletely understood. We found that *MUC16* was significantly upregulated in LUAD and correlated with poor prognosis. *In vitro* experiments further showed that *MUC16* knockdown inhibited proliferation, migration, and invasion of lung adenocarcinoma cells while promoting apoptosis, whereas *MUC16* overexpression produced the opposite effects. Further single-gene GSEA of MUC16 showed that genes associated with high MUC16 expression were significantly enriched in PI3K/AKT-related signaling pathways. Our Western blot results further confirmed that *MUC16* knockdown reduced p-PI3K and p-AKT levels, whereas *MUC16* overexpression increased the phosphorylation levels of PI3K and AKT. Further treatment with Buparlisib, a PI3K inhibitor, significantly suppressed the activation of PI3K/AKT signaling induced by MUC16 overexpression. Consistent with these molecular changes, Buparlisib attenuated the MUC16 overexpression-induced increases in EdU incorporation, migration, and invasion, further suggesting that MUC16 may promote malignant phenotypes in LUAD, at least in part, through PI3K/AKT signaling.

Notably, the overexpression plasmid used in this study mainly contained the MUC16 C-terminal sequence. Previous studies have shown that MUC16 can retain a membrane-bound C-terminal fragment (MUC16-C) after proteolytic cleavage at the cell surface. This fragment has important signal-transducing functions and may be involved in the activation of downstream pathways, including AKT, by interacting with membrane-proximal signaling molecules or kinase complexes (33, 34). Our additional experiments further identified FAK as a potential mediator of this process. Reciprocal Co-IP assays showed that MUC16 and FAK were present in the same protein complex, while immunofluorescence analysis revealed their spatial colocalization. Moreover, *MUC16* silencing reduced, whereas *MUC16* overexpression increased, FAK phosphorylation in both PC9 and H1975 cells. Pharmacological inhibition of FAK with defactinib attenuated the *MUC16* overexpression-induced increase in FAK phosphorylation, accompanied by corresponding reductions in PI3K and AKT phosphorylation, and partially reversed the *MUC16* overexpression-induced enhancement of migration and invasion in PC9 cells. Collectively, these findings suggest that MUC16 may promote malignant phenotypes in LUAD cells and contribute to LUAD progression, at least in part, through FAK-mediated activation of PI3K/AKT signaling.

Although multiple public datasets and *in vitro* assays supported our findings, further work is still needed. In particular, the prognostic signature should be verified in larger clinical cohorts. Moreover, immune features were mainly inferred computationally, while the small GSE126044 cohort provided only preliminary clinical support; validation in larger immunotherapy cohorts is therefore warranted. Further studies, particularly *in vivo* experiments, are needed to further validate the role of FAK in MUC16-mediated PI3K/AKT activation.

In conclusion, we established and validated an EGRG-related prognostic signature for LUAD with favorable predictive performance and identified *MUC16* as a potential key gene promoting LUAD progression. Functional and mechanistic experiments suggested that MUC16 may promote malignant phenotypes in LUAD cells, at least in part, through FAK-mediated activation of PI3K/AKT signaling. These results offer novel insights into LUAD risk stratification, prognostic assessment, and the exploration of potential therapeutic targets.

## Data Availability

The original contributions presented in the study are included in the article/**Supplementary Material**. Further inquiries can be directed to the corresponding author.
