# Supplementary material for: An endoplasmic reticulum stress- and Golgi apparatus-related signature reveals immune microenvironment remodeling and MUC16-driven PI3K/AKT activation in lung adenocarcinoma
Source: Front Immunol. 2026 Sep 8;17:1887786. doi: 10.3389/fimmu.2026.1887786 (PMC13593886; doi:10.3389/fimmu.2026.1887786)
Supplement: Supplementary file 1 [file SupplementaryFile1.docx]

**Supplementary Table**

**Supplementary Table 1*.*** **SiRNAs sequences used in MUC16 knockdown experiment.**

| Name | Sense or antisense | siRNA sequence |
| --- | --- | --- |
| MUC16 siRNA-1 | sense sequence | 5ʹ- AAGAAAAUGUGGUUUUGACUC-3ʹ |
|  | antisense sequence | 5ʹ- GUCAAAACCACAUUUUCUUCA-3ʹ |
| MUC16 siRNA-2 | sense sequence | 5ʹ- AUGUUAAAUGAUAGACAUCAU-3ʹ |
|  | antisense sequence | 5ʹ- GAUGUCUAUCAUUUAACAUCC-3ʹ |
| MUC16 siRNA-3 | sense sequence | 5ʹ- UGAAAAAGGCAAUGUUAAGCU-3ʹ |
|  | antisense sequence | 5ʹ- CUUAACAUUGCCUUUUUCAUC-3ʹ |

Supplementary Table 2: Comparative C-index performance of 101 machine-learning models in the TCGA-LUAD training cohort and two external GEO validation cohorts.

| Model（Rank） | TCGA-LUAD | GSE72094 | GSE30219 | Mean C-index |
| --- | --- | --- | --- | --- |
| RSF | 0.939 | 0.654 | 0.728 | 0.774 |
| Lasso + RSF | 0.944 | 0.654 | 0.666 | 0.755 |
| CoxBoost + RSF | 0.943 | 0.650 | 0.672 | 0.755 |
| StepCox[both] + RSF | 0.933 | 0.643 | 0.661 | 0.746 |
| StepCox[backward] + RSF | 0.933 | 0.643 | 0.661 | 0.746 |
| GBM | 0.830 | 0.671 | 0.669 | 0.724 |
| RSF + GBM | 0.791 | 0.664 | 0.701 | 0.719 |
| Lasso + GBM | 0.821 | 0.656 | 0.636 | 0.704 |
| CoxBoost + GBM | 0.824 | 0.647 | 0.635 | 0.702 |
| StepCox[both] + GBM | 0.800 | 0.649 | 0.629 | 0.693 |
| StepCox[backward] + GBM | 0.800 | 0.649 | 0.629 | 0.693 |
| RSF + survival-SVM | 0.670 | 0.685 | 0.709 | 0.688 |
| RSF + SuperPC | 0.664 | 0.677 | 0.709 | 0.683 |
| CoxBoost + survival-SVM | 0.680 | 0.677 | 0.673 | 0.677 |
| RSF + Enet[α=0.1] | 0.672 | 0.665 | 0.671 | 0.669 |
| Lasso + survival-SVM | 0.679 | 0.674 | 0.654 | 0.669 |
| RSF + Enet[α=0.2] | 0.672 | 0.663 | 0.668 | 0.668 |
| RSF + Ridge | 0.672 | 0.670 | 0.663 | 0.668 |
| RSF + CoxBoost | 0.671 | 0.658 | 0.672 | 0.667 |
| RSF + Enet[α=0.3] | 0.672 | 0.663 | 0.666 | 0.667 |
| survival - SVM | 0.667 | 0.675 | 0.658 | 0.667 |
| RSF + Enet[α=0.4] | 0.672 | 0.662 | 0.665 | 0.666 |
| RSF + Enet[α=0.5] | 0.672 | 0.661 | 0.664 | 0.666 |
| RSF + Enet[α=0.6] | 0.671 | 0.661 | 0.663 | 0.665 |
| RSF + Enet[α=0.7] | 0.671 | 0.660 | 0.663 | 0.665 |
| RSF + Enet[α=0.8] | 0.671 | 0.660 | 0.663 | 0.665 |
| RSF + StepCox[forward] | 0.672 | 0.653 | 0.671 | 0.665 |
| CoxBoost + SuperPC | 0.665 | 0.650 | 0.681 | 0.665 |
| RSF + Enet[α=0.9] | 0.671 | 0.660 | 0.662 | 0.664 |
| RSF + Lasso | 0.671 | 0.660 | 0.663 | 0.664 |
| RSF + plsRcox | 0.672 | 0.654 | 0.665 | 0.664 |
| Enet[α=0.1] | 0.695 | 0.663 | 0.603 | 0.654 |
| RSF + StepCox[both] | 0.661 | 0.657 | 0.640 | 0.653 |
| RSF + StepCox[backward] | 0.661 | 0.657 | 0.640 | 0.653 |
| StepCox[both] + survival-SVM | 0.675 | 0.676 | 0.609 | 0.653 |
| StepCox[backward] + survival-SVM | 0.675 | 0.676 | 0.609 | 0.653 |
| Lasso + SuperPC | 0.664 | 0.650 | 0.646 | 0.653 |
| CoxBoost + Ridge | 0.691 | 0.654 | 0.607 | 0.650 |
| CoxBoost | 0.689 | 0.658 | 0.599 | 0.649 |
| Enet[α=0.2] | 0.694 | 0.658 | 0.592 | 0.648 |
| CoxBoost + Enet[α=0.1] | 0.691 | 0.653 | 0.599 | 0.648 |
| Enet[α=0.3] | 0.693 | 0.657 | 0.590 | 0.647 |
| Enet[α=0.4] | 0.694 | 0.656 | 0.591 | 0.647 |
| Lasso + CoxBoost | 0.690 | 0.656 | 0.596 | 0.647 |
| CoxBoost + Enet[α=0.2] | 0.691 | 0.651 | 0.594 | 0.646 |
| Enet[α=0.5] | 0.693 | 0.655 | 0.588 | 0.645 |
| Enet[α=0.6] | 0.693 | 0.654 | 0.588 | 0.645 |
| Enet[α=0.7] | 0.692 | 0.654 | 0.589 | 0.645 |
| Enet[α=0.8] | 0.692 | 0.654 | 0.589 | 0.645 |
| Enet[α=0.9] | 0.692 | 0.654 | 0.589 | 0.645 |
| CoxBoost + Enet[α=0.3] | 0.691 | 0.651 | 0.593 | 0.645 |
| Lasso | 0.692 | 0.654 | 0.588 | 0.645 |
| CoxBoost + Enet[α=0.4] | 0.691 | 0.650 | 0.593 | 0.644 |
| CoxBoost + Enet[α=0.5] | 0.690 | 0.649 | 0.590 | 0.643 |
| CoxBoost + Enet[α=0.6] | 0.690 | 0.649 | 0.589 | 0.643 |
| Ridge | 0.705 | 0.627 | 0.597 | 0.643 |
| CoxBoost + Enet[α=0.7] | 0.690 | 0.649 | 0.588 | 0.642 |
| CoxBoost + Enet[α=0.8] | 0.689 | 0.649 | 0.588 | 0.642 |
| CoxBoost + Enet[α=0.9] | 0.689 | 0.649 | 0.588 | 0.642 |
| CoxBoost + Lasso | 0.689 | 0.649 | 0.587 | 0.642 |
| CoxBoost + plsRcox | 0.690 | 0.644 | 0.584 | 0.639 |
| Lasso + plsRcox | 0.692 | 0.649 | 0.577 | 0.639 |
| StepCox[both] + Enet[α=0.1] | 0.682 | 0.654 | 0.577 | 0.638 |
| StepCox[both] + Ridge | 0.683 | 0.655 | 0.578 | 0.638 |
| StepCox[backward] + Enet[α=0.1] | 0.682 | 0.654 | 0.577 | 0.638 |
| StepCox[backward] + Ridge | 0.683 | 0.655 | 0.578 | 0.638 |
| CoxBoost + StepCox[forward] | 0.689 | 0.645 | 0.581 | 0.638 |
| Lasso + StepCox[forward] | 0.690 | 0.649 | 0.573 | 0.638 |
| StepCox[both] + CoxBoost | 0.681 | 0.653 | 0.578 | 0.637 |
| StepCox[both] + Enet[α=0.2] | 0.681 | 0.653 | 0.577 | 0.637 |
| StepCox[both] + Enet[α=0.3] | 0.682 | 0.653 | 0.578 | 0.637 |
| StepCox[both] + Enet[α=0.4] | 0.682 | 0.653 | 0.577 | 0.637 |
| StepCox[backward] + CoxBoost | 0.681 | 0.653 | 0.578 | 0.637 |
| StepCox[backward] + Enet[α=0.2] | 0.681 | 0.653 | 0.577 | 0.637 |
| StepCox[backward] + Enet[α=0.3] | 0.682 | 0.653 | 0.578 | 0.637 |
| StepCox[backward] + Enet[α=0.4] | 0.682 | 0.653 | 0.577 | 0.637 |
| StepCox[both] + Enet[α=0.5] | 0.681 | 0.651 | 0.576 | 0.636 |
| StepCox[both] + Enet[α=0.6] | 0.681 | 0.652 | 0.576 | 0.636 |
| StepCox[both] + Enet[α=0.7] | 0.681 | 0.652 | 0.576 | 0.636 |
| StepCox[both] + Enet[α=0.8] | 0.681 | 0.652 | 0.576 | 0.636 |
| StepCox[both] + Enet[α=0.9] | 0.681 | 0.651 | 0.576 | 0.636 |
| StepCox[both] + Lasso | 0.681 | 0.652 | 0.576 | 0.636 |
| StepCox[backward] + Enet[α=0.5] | 0.681 | 0.651 | 0.576 | 0.636 |
| StepCox[backward] + Enet[α=0.6] | 0.681 | 0.652 | 0.576 | 0.636 |
| StepCox[backward] + Enet[α=0.7] | 0.681 | 0.652 | 0.576 | 0.636 |
| StepCox[backward] + Enet[α=0.8] | 0.681 | 0.652 | 0.576 | 0.636 |
| StepCox[backward] + Enet[α=0.9] | 0.681 | 0.651 | 0.576 | 0.636 |
| StepCox[backward] + Lasso | 0.681 | 0.652 | 0.576 | 0.636 |
| StepCox[both] | 0.681 | 0.650 | 0.574 | 0.635 |
| StepCox[backward] | 0.681 | 0.650 | 0.574 | 0.635 |
| StepCox[both] + plsRcox | 0.681 | 0.650 | 0.573 | 0.635 |
| StepCox[backward] + plsRcox | 0.681 | 0.650 | 0.573 | 0.635 |
| StepCox[forward] | 0.703 | 0.613 | 0.585 | 0.634 |
| CoxBoost + StepCox[both] | 0.683 | 0.654 | 0.563 | 0.633 |
| CoxBoost + StepCox[backward] | 0.683 | 0.654 | 0.563 | 0.633 |
| plsRcox | 0.703 | 0.611 | 0.584 | 0.633 |
| Lasso + StepCox[both] | 0.683 | 0.654 | 0.563 | 0.633 |
| Lasso + StepCox[backward] | 0.683 | 0.654 | 0.563 | 0.633 |
| StepCox[both] + SuperPC | 0.614 | 0.650 | 0.634 | 0.632 |
| StepCox[backward] + SuperPC | 0.614 | 0.650 | 0.634 | 0.632 |
| SuperPC | 0.642 | 0.622 | 0.623 | 0.629 |

Supplementary Table 3*.* Comparison of prognostic significance and correlations with the overall risk score among CFTR, SLC2A1, and MUC16

| **Gene** | **HR** | **Lower 95% CI** | **Upper 95% CI** | **Cox P** | **Spearman ρ** | **Correlation P** |
| --- | --- | --- | --- | --- | --- | --- |
| **CFTR** | 0.782 | 0.667 | 0.918 | 2.63 × 10⁻³ | −0.244 | 9.88 × 10⁻⁸ |
| **SLC2A1** | 1.525 | 1.294 | 1.796 | 4.53 × 10⁻⁷ | 0.507 | 1.04 × 10⁻³¹ |
| **MUC16** | 1.193 | 1.028 | 1.384 | 2.01 × 10⁻² | 0.272 | 2.73 × 10⁻⁹ |
