# Supplementary material for: An endoplasmic reticulum stress- and Golgi apparatus-related signature reveals immune microenvironment remodeling and MUC16-driven PI3K/AKT activation in lung adenocarcinoma
Source: Front Immunol. 2026 Sep 8;17:1887786. doi: 10.3389/fimmu.2026.1887786 (PMC13593886; doi:10.3389/fimmu.2026.1887786)
Supplement: Supplementary file 2 [file SupplementaryFile2.docx]

**Supplementary Figures**

*Supplementary Figure 1.* **GSEA of EGR-DEGs in lung adenocarcinoma.** (A) GSEA illustrating representative activated biological processes. (B) GSEA illustrating representative inhibited biological processes.

*Supplementary Figure 2*. Distribution of risk scores, survival status, and model-gene expression in the TCGA-LUAD, GSE72094, and GSE30219 cohorts. (A) TCGA-LUAD cohort. (B) GSE72094 cohort. (C) GSE30219 cohort. In each cohort, the upper panel shows the distribution of risk scores, the middle panel shows patient survival status, and the lower panel shows the expression patterns of the model genes across low- and high-risk groups.

*Supplementary Figure 3.* External validation of the 17-gene prognostic signature. (A, B) Kaplan–Meier survival curves in the GSE26939 and GSE31210 cohorts. (C, D) Time-dependent ROC curves for 1-, 3-, and 5-year overall survival. (E, F) Risk score distribution, survival status, and expression heatmaps of the 17 signature genes in the two external cohorts.

*Supplementary Figure 4*. Subgroup analysis of survival and risk score. (A, B) Kaplan-Meier curves in female and male subgroups. (C, D) Kaplan-Meier curves in age subgroups (≤65 and >65 years). (E, F) Kaplan-Meier curves in T1–2 and T3–4 subgroups. (G, H) Kaplan-Meier curves in N0 and N1–3 subgroups. (I, J) Kaplan-Meier curves in M0 and M1 subgroups. (K, L) Kaplan-Meier curves in stage I–II and stage III–IV subgroups. (M–R) Risk score distribution across sex, T stage, age, N stage, M stage, and clinical stage subgroups.

*Supplementary Figure 5.* Comparison of RSF-predicted risk scores between responders and non-responders in the GSE126044 immunotherapy cohort. The established 17-gene RSF model was applied to 16 patients with NSCLC treated with anti-PD-1 therapy, including 5 responders and 11 non-responders. Non-responders tended to have higher predicted risk scores than responders, although the difference was not statistically significant. Each dot represents an individual patient; boxes indicate the median and interquartile range. Statistical significance was assessed using the Wilcoxon rank-sum test.

*Supplementary Figure 6*. Tumor mutation burden in the low- and high-risk groups. (A) Comparison of TMB between the two risk groups. (B) Correlation between TMB and risk score. (C) Kaplan-Meier curves based on combined TMB and risk stratification. (D) Mutation landscape of the high-risk group. (E) Mutation landscape of the low-risk group.

*Supplementary Figure 7*. Protein-protein interaction network of the 17 model genes constructed using STRING.

*Supplementary Figure 8.* Single-gene gene set enrichment analysis of MUC16-associated signaling pathways. (A) Enrichment of the GO biological process phosphatidylinositol 3-kinase/protein kinase B signal transduction. (B) Enrichment of the Hallmark PI3K–AKT–mTOR signaling pathway. The normalized enrichment score (NES) and adjusted P value are shown in each panel.

*Supplementary Figure 9* Determination of Buparlisib IC50 values for pathway inhibition experiments. (A, B) MUC16-overexpressing PC9 and H1975 cells were treated with increasing concentrations of Buparlisib, and cell viability was assessed using CCK-8 assays. The calculated IC50 values were 2.692 μM for PC9 cells and 1.608 μM for H1975 cells. These values were used to determine sub-IC50 concentrations for subsequent PI3K/AKT pathway inhibition experiments.
